# Identification and Dissection of Four Major QTL Affecting Milk Fat Content in the German Holstein-Friesian Population

**DOI:** 10.1371/journal.pone.0040711

**Published:** 2012-07-11

**Authors:** Xiaolong Wang, Christine Wurmser, Hubert Pausch, Simone Jung, Friedrich Reinhardt, Jens Tetens, Georg Thaller, Ruedi Fries

**Affiliations:** 1 Chair of Animal Breeding, Technische Universitaet Muenchen, Freising-Weihenstephan, Germany; 2 Vereinigte Informationssysteme Tierhaltung w.V., Verden/Aller, Germany; 3 Institute of Animal Breeding and Husbandry, Christian-Albrechts-Universitaet, Kiel, Germany; University of South Florida College of Medicine, United States of America

## Abstract

Milk composition traits exhibit a complex genetic architecture with a small number of major quantitative trait loci (QTL) explaining a large fraction of the genetic variation and numerous QTL with minor effects. In order to identify QTL for milk fat percentage (FP) in the German Holstein-Friesian (HF) population, a genome-wide association study (GWAS) was performed. The study population consisted of 2327 progeny-tested bulls. Genotypes were available for 44,280 SNPs. Phenotypes in the form of estimated breeding values (EBVs) for FP were used as highly heritable traits. A variance components-based approach was used to account for population stratification. The GWAS identified four major QTL regions explaining 46.18% of the FP EBV variance. Besides two previously known FP QTL on BTA14 (*P* = 8.91×10−^198^) and BTA20 (*P* = 7.03×10^−12^) within *DGAT1* and *GHR*, respectively, we uncovered two additional QTL regions on BTA5 (*P* = 2.00×10^−13^) and BTA27 (*P* = 9.83×10^−5^) encompassing *EPS8* and *GPAT4*, respectively. *EPS8* and *GPAT4* are involved in lipid metabolism in mammals. Re-sequencing of *EPS8* and *GPAT4* revealed 50 polymorphisms. Genotypes for five of them were inferred for the entire study population. Two polymorphisms affecting potential transcription factor binding sites of *EPS8* (*P* = 1.40×10^−12^) and *GPAT4* (*P* = 5.18×10^−5^), respectively, were highly significantly associated with the FP EBV. Our results provide evidence that alteration of regulatory sites is an important aspect of genetic variation of complex traits in cattle.

## Introduction

Improvement of milk yield and composition is a major objective of dairy cattle breeding programs and highly reliable breeding values are estimated to this end. Milk composition traits such as protein and fat content are not only important production traits but also permit insights into the metabolic constitution of lactating cows [1].

Milk fat content, indicated as fat percentage (FP), is a prototypical complex quantitative trait determined by numerous loci with small effects and only few loci with major effects [2]. Family-based linkage studies and genome-wide association studies (GWAS) have already identified several genomic regions contributing to the genetic variation of FP in cattle (*e.g.* [2], [3], [4], [5]). Among them, most prominently, a K232A-substitution within the acyl-CoA:diacylglycerol acyltransferase encoding gene *DGAT1* [6], [7] and a F279Y-substitution within the growth hormone receptor encoding gene *GHR* [8], [9] have been well characterized. These two polymorphisms account for a major fraction of the genetic variation of FP in various cattle breeds (*e.g.* [10], [11], [12]).

The aim of the present study was to identify major quantitative trait loci (QTL) for FP in the German HF population by genome-wide association analysis and to pinpoint the causal variants. The study revealed four QTL, two of them novel. We report candidate genes and putative causal variants for the newly identified QTL.

## Results

### Genotypes and Quality Control

The HF bulls were genotyped with the Illumina Bovine SNP 50 K BeadChip® comprising 54,001 single nucleotide polymorphisms (SNPs). Of 2401 genotyped animals, 62 were removed from the data set because genotyping failed for more than 10% of the SNPs. Twelve animals showed major differences between pedigree and genomic relationship and were excluded from further analysis (Figure S1). 549 SNPs with unknown chromosomal position and 7951 SNPs with minor allele frequencies <0.01 were omitted from subsequent analyses. 732 SNPs that were missing in more than 10% of the animals and 966 SNPs that deviated significantly from the Hardy-Weinberg equilibrium (*P*<0.001) were excluded. The final data set comprised 2327 animals and 44,280 SNPs.

### Association study

**Figure 1:**
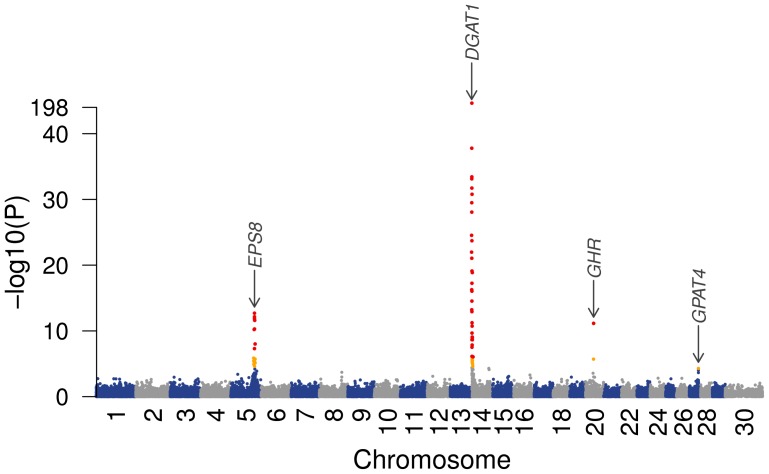
Association of 44,280 SNPs with the estimated breeding values for milk fat percentage in 2327 animals of the German Holstein-Friesian population. Red symbols represent SNPs with *P*<1.3×10^−6^ (Bonferroni-corrected significance level), orange symbols indicate chromosome-wide significance.

The genome-wide association study based on 44,280 SNPs and 2327 progeny-tested bulls identified four QTL for FP on BTA5, BTA14, BTA20 and BTA27 in the German HF population (**Figure 1**).

**Figure 2:**
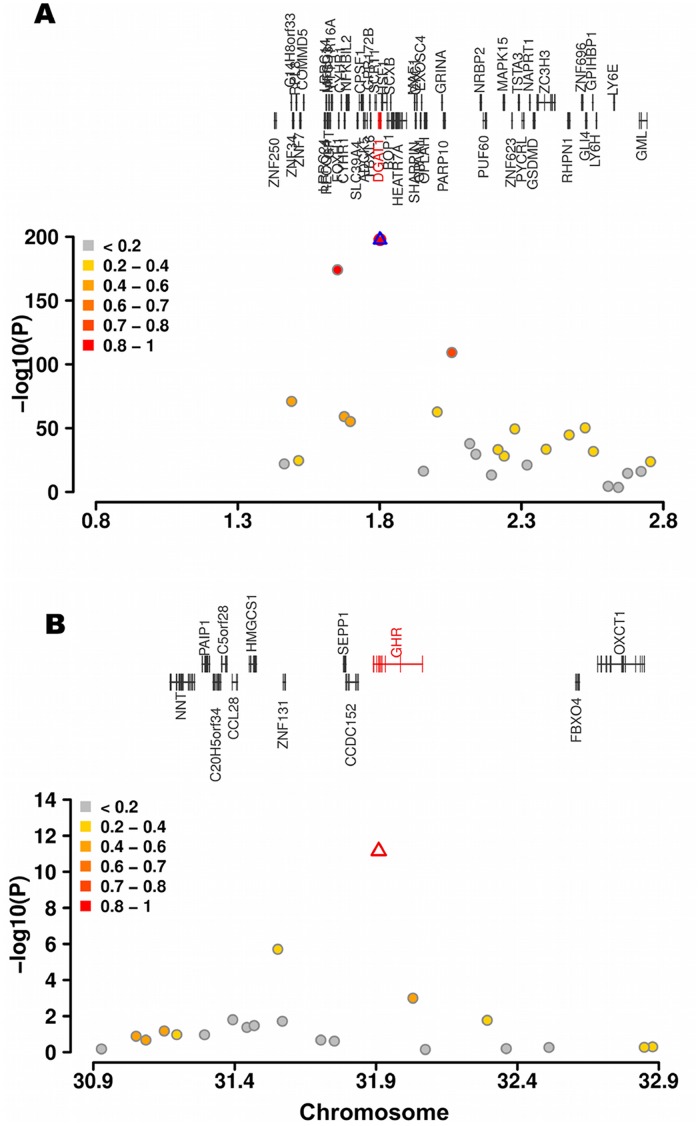
Detailed view of two genomic regions within known QTL for milk fat content in cattle. QTL regions on BTA14 (**A**) and BTA20 (**B**) encompassing *DGAT1* and *GHR*, respectively. Open symbols (blue and red triangles) represent the K232A- and the F279Y-substitution, respectively. Different colors indicate the extent of linkage disequilibrium (r^2^) between the postulated QTN and all other SNPs.

Forty-eight significantly associated SNPs encompass the DGAT1 encoding gene (1.46 Mb –7.31 Mb) and define the QTL region on BTA14. The most significantly associated SNP (*P* = 1.57×10^−198^) resides 1149 bp upstream of the postulated *DGAT1*-QTN [6], [7]. Genotyping of the K232A-substitution within *DGAT1* showed close to complete linkage disequilibrium with the SNP ARS-BFGL-NGS-4939 (r^2^ = 0.998). The *P*-value for the latter SNP is marginally lower than for the K232A marker due to the imputed genotypes of two animals, probably indicating inaccurate imputation rather than incomplete linkage disequilibrium **(** **Figure 2A** **)**.

On BTA20, a single SNP (BTA-84181-no-rs) located 357 kb downstream of the postulated causal F279Y-substitution within the GHR encoding gene [9] was significantly associated (*P* = 1.95×10^−6^). The association signal for the F279Y-substitution was more prominent than for any other SNP on BTA20 (*P* = 7.03×10^−12^) **(** **Figure 2B** **)**. BTA-84181-no-rs SNP is only moderately linked with the F279Y-substitution (r^2^ = 0.2). We additionally obtained genotypes for the S18N-substitution within the PRLR encoding gene [8], another presumed causal variant. The S18N-substitution is 7.56 Mb distant from the significantly associated SNP BTA-84181-no-rs. No association of the *PRLR-*variant (*P* = 0.319) with the FP EBV was observed.

**Figure 3:**
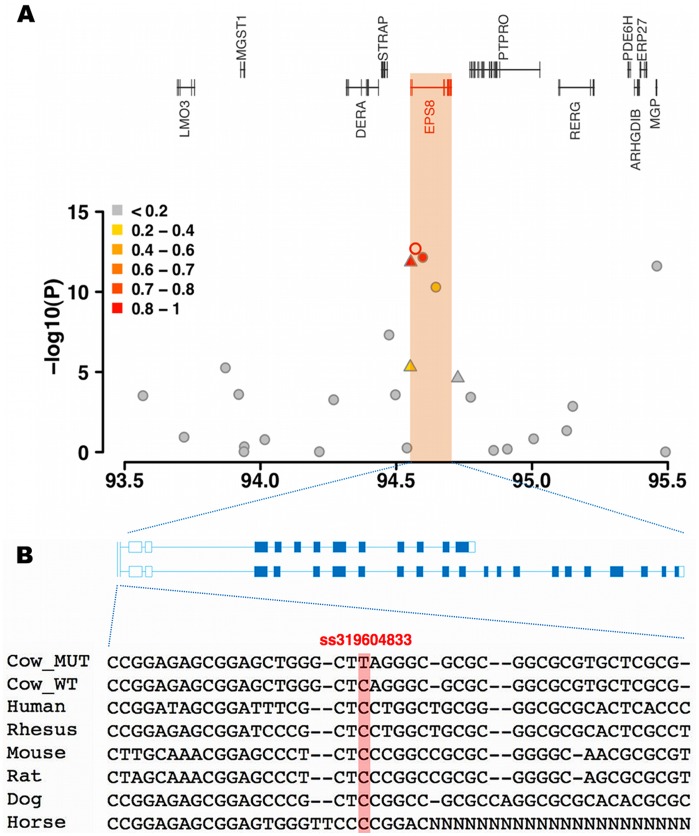
Schematic view of the BTA5 QTL region encompassing *EPS8*. (**A**) The open symbol represents the most significantly associated SNP. Different colors indicate the extent of linkage disequilibrium (r^2^) between the most significantly associated SNP and all other SNPs. Triangles and circles indicate SNPs resulting from re-sequencing and genotyping with the 50 K BeadChip, respectively. (**B**) Two alternative transcripts of *EPS8* are present in cattle. The multispecies sequence alignment of a segment in the promoter region of *EPS8* encompassing the highly significantly associated ss319604833 polymorphism (red background) illustrates the high conservation among species.

Seven genome-wide significantly associated SNPs located between 91.2 Mb and 97.1 Mb delineate the QTL region on BTA5. The most significantly associated SNP Hapmap49734-BTA-74577 (*P* = 2.00×10^−13^) is located in the second intron of the epidermal growth factor receptor pathway substrate 8 encoding gene (*EPS8)* (**Table 1**) **(** **Figure 3** **)**.

**Table 1:** Characteristics of the most significantly associated 50 K Illumina BeadChip SNPs and additional polymorphisms of four major QTL for milk fat percentage in the German Holstein-Friesian population.

SNP	BTA	Physical position a	Minor allele (MAF)	*P* b	Neighboring gene
Hapmap49734-BTA-74577	5	94,570,828	A (0.09)	2.00×10^−13^	*EPS8*
ARS-BFGL-NGS-4939	14	1,801,116	G (0.31)	1.57×10^−198^	*DGAT1*
BTA-84181-no-rs	20	31,552,475	G (0.37)	1.95×10^−6^	*GHR*
ARS-BFGL-NGS-57448	27	36,155,097	A (0.36)	9.83×10^−5^	*GPAT4*
ss319604831	5	94,551,792	G (0.21)	4.92×10^−6^	*EPS8*
ss319604833	5	94,553,580	T (0.09)	1.40×10^−12^	*EPS8*
ss319604845	5	94,726,848	T (0.22)	2.40×10^−5^	*EPS8*
K232A	14	1,802,265	A (0.31)	8.91×10^−198^	*DGAT1*
F279Y	20	31,909,479	A (0.17)	7.03×10^−12^	*GHR*
S18N	20	39,115,344	G (0.14)	3.19×10^−1^	*PRLR*
ss410759404	27	36,211,257	GA (0.38)	5.18×10^−5^	*GPAT4*
ss410758894	27	36,228,939	A (0.40)	2.27×10^−4^	*GPAT4*

a The SNPs are ordered according to their position on the UMD3.1 assembly of the bovine genome sequence.

b The *P*-values are obtained after regression analysis and by using a variance components based approach to account for population stratification.

**Figure 4:**
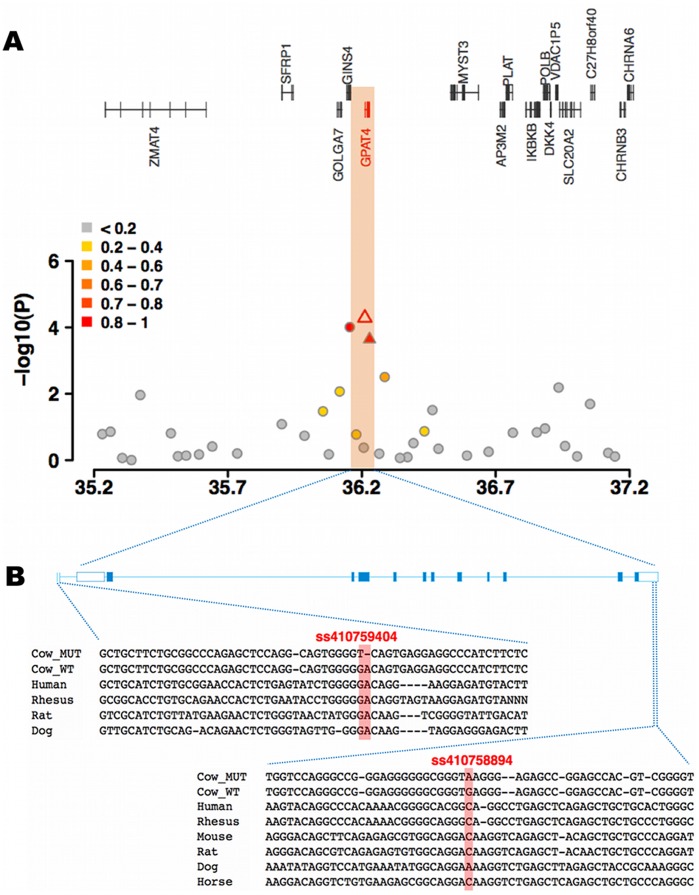
Schematic view of the BTA27 QTL region encompassing *GPAT4*. (**A**) The open symbol represents the most significantly associated SNP. Different colors indicate the extent of linkage disequilibrium (r^2^) between the most significantly associated SNP and all other SNPs. The triangles indicate SNPs resulting from re-sequencing, circles indicate SNPs from genotyping with the 50 K BeadChip. (**B**) Gene structure of *GPAT4* and multispecies sequence alignment of the promoter encompassing the highly significantly associated ss410759404 and of the 3′UTR encompassing the highly significantly associated ss410758894, respectively.

On BTA27, SNP ARS-BFGL-NGS-57448 is associated with *P* = 9.83×10^−5^ and thus with chromosome-wide significance only. It is located about 56 kb upstream of the glycerol-3-phosphate acyltransferase 4 encoding gene (*GPAT4)* (**Table1) (** **Figure 4** **)**.

### Assessing the Impact of the Four QTL

**Figure 5:**
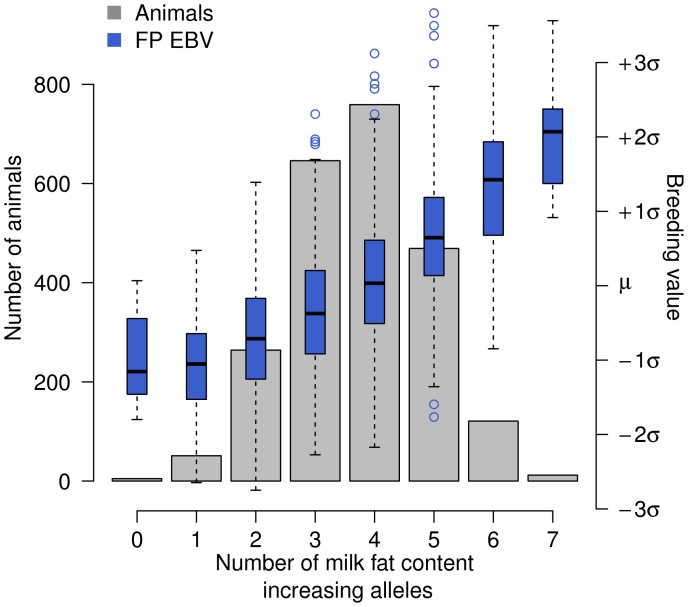
The combined impact of the four identified QTL on BTA5, 14, 20 and 27 on the estimated breeding value for milk fat percentage in the German Holstein-Friesian population. 2327 Holstein-Friesian animals are grouped according to the number of alleles that increase the milk FP EBV. The grey bars indicate the number of animals with an increasing number of FP increasing alleles. The box plots represent the FP EBVs for each group. No animals carrying eight FP increasing alleles were observed in the study population.

Alleles increasing the FP EBVs were identified for the most significantly associated SNP for each of the four QTL regions. The frequency distribution of animals with an increasing number of alleles is displayed in **Figure 5**. The EBVs of animals with one and seven FP increasing alleles differ by more than three standard deviations.

### Chromosomal Partitioning of the EBV Variance

**Figure 6:**
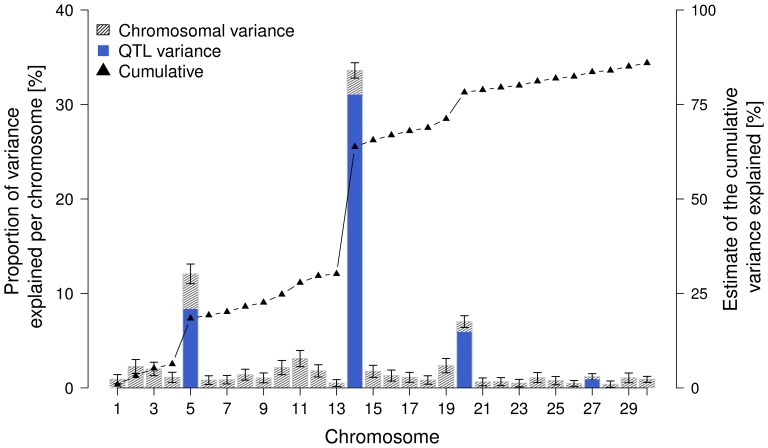
Partitioning of the genetic variance onto 30 chromosomes and four identified QTL regions on BTA5, 14, 20 and 27. The grey shaded bars indicate the fraction of EBV variance attributed to a particular chromosome and the corresponding standard error. The dark grey bars represent the fraction of EBV variance attributed to each of the four identified QTL regions. The black triangles represent the cumulative proportion of EBV variance explained.

The proportion of the EBV variance attributed to a particular chromosome/QTL was estimated with the effects of all chromosomes/QTL fitted simultaneously. Totally, the 44,280 SNPs account for 85.97% of the EBV variance. The contribution of particular chromosomes varies strongly (**Figure 6**). A major fraction of the EBV variance is attributable to BTA14 (33.60%), BTA5 (12.08%) and BTA20 (7.01%). BTA27 accounts for a minor fraction (1.19%) of the EBV variance only. Totally, the four identified QTL explain 46.18% of the FP EBV variance. The estimates of the EBV variance attributable to the four QTL are 8.35% (BTA5), 31.04% (BTA14), 5.91% (BTA20) and 0.88% (BTA27).

### Molecular-genetic Analysis of the BTA5 QTL

*EPS8* was considered as a positional candidate gene for the FP QTL region on BTA5 as the two most significantly associated SNPs were located within its second intron. *EPS8* plays a role in the fat metabolism of mammals as it is a substrate for the EGFR-kinase [13]. The EGFR-kinase activates transcriptional regulators of fatty acid synthesis and thereby increases the concentration of intracellular fatty acids [14]. Re-sequencing of *EPS8* in 24 animals resulted in the identification of 20 polymorphisms (**Table S4**). We genotyped two promoter polymorphisms (ss319604831 and ss319604833) in high linkage disequilibrium (r^2^>0.8) with SNP Hapmap49734-BTA-74577 and for one non-synonymous mutation (M599T, ss319604845) located in a highly conserved region of *EPS8* (**Table 1** **,** **Figure 3**). However, only ss319604833, located 100 bp upstream of the transcription start of *EPS8*, was highly associated with the FP EBV (*P* = 1.40×10^−12^) (**Table 1**) **(** **Figure 3** **)**. The significance level of the putative causal variant ss319604833 was marginally lower compared to the SNP Hapmap49734-BTA-74577 (*P* = 2.00×10^−13^). This could be due to imperfect imputation, since 9.2% of the genotypes were missing after genotyping and thus were imputed.

**Figure 7:**
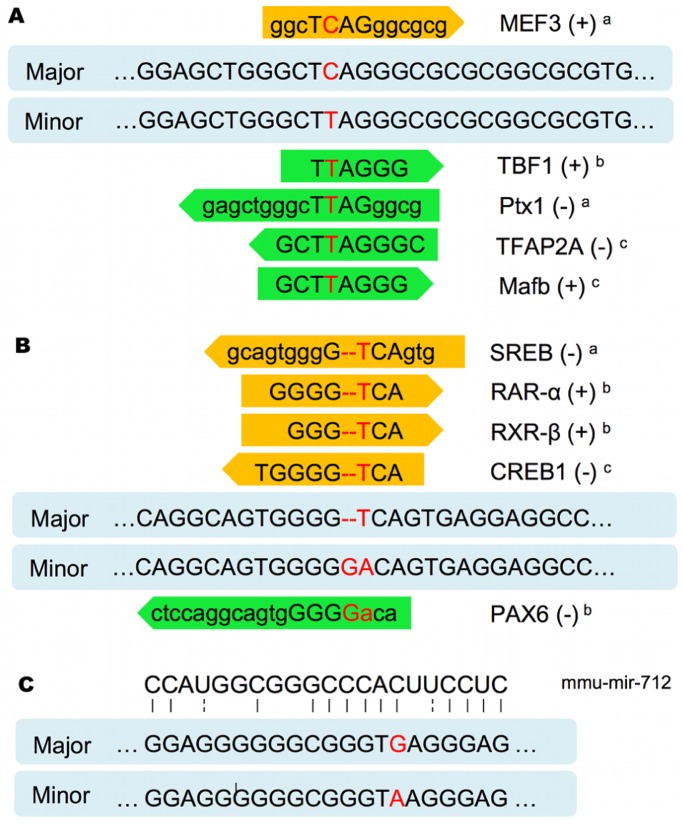
Prediction of regulatory sites for the polymorphisms in *EPS8* and *GPAT4*. Prediction of transcription factor binding sites within the promoter sequences of *EPS8* and *GPAT4* encompassing the SNP ss319604833 (**A**) and the SNP ss410759404 (**B**). (+) and (−) indicate forward and reverse direction. *MatInspector^a^, TESS^b^* and *JASPAR^c^* were used for the prediction of transcription factor binding sites. (**C**) Prediction of a miRNA binding site for mmu-mir-712 (free energy: −27.93 kcal/mol) within the 3′UTR of *GPAT4* encompassing the polymorphism ss410758894.

ss319604833 is located in a region of *cis*-acting regulatory elements. Five transcription factors whose binding may be affected by the polymorphism were predicted. The promoter variant with the more frequent C allele, associated with a lower FP EBV, contains a potential MEF3-element. In contrast, TBF1-, Ptx1-, MafB- and TFAP2A-elements were predicted for the sequence with the rare T allele, associated with an increased FP EBV **(** **Figure 7A** **)**. The expression of transcription factor TFAP2A is significantly correlated with the concentration of nonesterified fatty acids (NEFA) and liver triacylglycerol [15].

### Molecular-genetic Analysis of the BTA27 QTL

The FP QTL region on BTA27 encompasses the *GPAT4* gene. *GPAT4* encodes the rate-limiting enzyme glycerol-3-phosphate acyltransferase in the triacylglycerol biosynthesis pathway and plays a key role in milk fat biosynthesis [16]. Re-sequencing of the exons and regulatory flanking regions in a panel of twelve animals revealed 30 polymorphisms (**Table S4**). Genotypes for two polymorphisms (ss410759404 and ss410758894) located within the 5′flanking region and within the 3′UTR, respectively, were obtained for the entire study population (**Table 1**) **(** **Figure 4** **)**. ss410759404 (GA>T), was more strongly associated with the FP EBV (*P* = 5.18×10^−5^) than any other SNP on BTA27.

ss410759404 is located 1378 bp upstream of the translation start of *GPAT4*. Transcription factors SREB, CREB, RXR-α and RAR-β were predicted for the sequence with the frequent T allele, which is associated with a lower FP EBV. The PAX6-element was predicted for the sequence with the minor GA allele, that was associated with an increased FP EBV. **(** **Figure 7B** **)**. RXR-α up-regulates genes involved in fatty acid and lipid metabolism during the process of adipogenesis [17], CREB takes part in the regulation of gluconeogenesis [18] and SREB is considered to be one of the central regulation factors in milk fat synthesis [16].

The prediction of putative miRNA binding sites revealed that the binding of mmu-miR-712 might be affected by the ss410758894 polymorphism, with the minor A allele showing less similarity to the miRNA-binding site **(** **Figure 7C** **)**. However, no conserved sites for miRNA families could be identified due to low conservation between species. Also, the *P*-value for ss410758894 (*P* = 2.27×10^−4^) did not meet the significance level.

## Discussion

Our genome-wide association study was based on a medium-sized sample of the German HF population and on a dense SNP map. It revealed four major QTL for FP. The four identified QTL regions account for a large part of the EBV variance (46.18%). However, a large fraction of the EBV variance is attributable to chromosomes with no identified QTL, *e.g.* BTA11 and BTA19. The QTL region on BTA14 encompassing *DGAT1* accounts for 31.04% of the EBV variance which agrees with previous findings [10]. The QTL region on BTA20 encompassing the F279Y-substitution within the GHR encoding gene on BTA20 [9] accounts for 5.91% of the EBV variance. There was no evidence for a second FP QTL on BTA20 resulting from the S18N-substitution within the *PRLR* encoding gene [8]. We were able to identify two additional QTL regions for FP in the German HF population on BTA5 and BTA27 that together account for 9.23% of the EBV variance. Our findings support the proposed genetic architecture of FP with numerous loci with small effects and only few loci with larger effects [2].

The presence of a FP QTL on BTA5 agrees with findings in the Australian HF population [2]. We identified a highly significantly associated polymorphism in the promoter region of *EPS8* which is supposed to mediate the binding of TFAP2A and concomitantly the transcription rate of *EPS8*. EPS8 physically interacts with the epidermal growth factor receptor [13]. Recently it has been shown that sterol regulatory element-binding proteins (SREBPs) are regulated by the epidermal growth factor [19]. SREBPs control the expression of genes required for the uptake and synthesis of cholesterol, fatty acid and triglycerides. Thus, it seems likely that an enhanced transcription rate of *EPS8*, conferred by binding of TFAP2A, results in an increased milk fat biosynthesis in the lactating mammary gland. Therefore, a contribution of ss319604833 to the genetic variation of milk fat synthesis seems plausible.

Recently, Bouwman and colleagues [20] reported a QTL contributing to the genetic variation of milk fatty acid composition in the Dutch HF population nearby *GPAT4,* supporting our findings of a FP QTL in the German HF population on BTA27. *GPAT4* plays a crucial role in lipid biosynthesis in mammals [16]. The transcription rate of *GPAT4* is highly correlated with the concentration of milk diacylglycerols and triacylglycerols [21], [22]. Prediction of transcription factor binding sites for the highly significantly associated SNP ss410759404 suggests that the binding of transcription factors involved in fat metabolism might be affected by this variant [16], [17], [18]. Hence, a contribution of ss410759404 to the milk fat biosynthesis capacity in lactating cows seems likely. Association analysis uncovered a second SNP (ss410758894) affecting a potential miRNA binding site of *GPAT4*. Although ss410758894 did not meet the criteria for significant association, it is possible that both variants contribute to the genetic variation of the BTA27 FP QTL in the German HF population.

## Materials and Methods

### Animals and Phenotypes

The study population consisted of 2401 progeny-tested Holstein Friesian bulls. The animals descend from 376 different sires and 423 maternal grand-sires. The paternal half-sib families and maternal grandsire families encompass up to 83 members with an average of six members. Phenotypes in the form of estimated breeding values (EBVs) for milk FP were obtained from vit w.V. Verden (www.vit.de, April 2010 version). Breeding value estimation for FP was carried out using best linear unbiased prediction (BLUP).

### Genome-wide Association Study

The HF bulls were genotyped with the Illumina Bovine SNP 50 K BeadChip® comprising 54,001 single nucleotide polymorphisms. The chromosomal positions of 53,452 SNPs were according to the University of Maryland UMD3.1 assembly of the bovine genome sequence [23]. SNPs with unknown position or if genotyping failed in more than 10% of the animals were excluded. SNPs with a minor allele frequency (MAF) <0.01 or significant (*P*<0.001) deviation from the Hardy-Weinberg equilibrium were omitted for subsequent analysis. Sporadically missing genotypes were imputed using default parameters of *Beagle* (version 3.2.1) [25]. Animals with more than 10% missing genotypes were not considered for further analyses. The genomic relationship of each pair of animals was obtained as proposed by VanRaden (2008) [24] and was compared with the corresponding pedigree relationship. Animals showing major inconsistencies between pedigree and genomic relationship were excluded from further analysis.

To account for population stratification, *EMMAX* [26] was used to fit the model 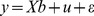, where y is a vector of EBVs for FP, b is the SNP effect, X is a design matrix of SNP genotypes, u is the random polygenic effect with (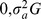), where 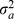 is the additive genetic variance, G is the genomic relationship matrix (GRM) among the 2327 animals (see above) and 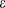 is the non heritable component of the random variation.

### Chromosomal Partitioning of the Genetic Variance

In order to estimate the proportion of EBV variance attributable to a particular chromosome and QTL, a GRM was built (see above) for each of the 30 chromosomes and the four QTL regions separately. A QTL was defined by the SNPs within a 5 Mb interval centered on the most significantly associated SNP. Chromosome-specific variance was estimated based on GRMs that included all SNPs on a chromosome except those within the 5 Mb QTL interval. We used *GCTA* [27] to fit the model 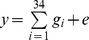, where y is a vector of EBVs for FP, g is a vector of genetic effects attributed to the i^th^ chromosome/QTL, and e is a vector of random residual deviates. g_i_ is assumed to be normally distributed with 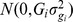, where G_i_ is the GRM built based on SNPs of the i^th^ chromosome/QTL. The proportion of variance attributable to the i^th^ chromosome/QTL was calculated as 
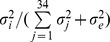
.

### Candidate Gene Annotation

The *GenomeThreader* software tool [28] was used to predict the genomic structure and localization of the genes based on the University of Maryland UMD3.1 assembly of the bovine genome sequence [23] and the Dana-Farber Cancer Institute bovine gene index release 12.0 [29] together with the annotated RNA sequences of the UMD3.1 assembly. The *GenomeThreader* output was viewed and edited using the *Apollo Genome Annotation and Curation Tool* [30]. The exon-intron organization of the annotated genes is summarized in **Table S1**. Transcription factor binding sites were predicted with *MatInspector* [31], *JASPAR* [32] and *TESS* [33]. Prediction of microRNAs was carried out using *MicroCosm Targets* (http://www.ebi.ac.uk/enright-srv/microcosm/htdocs/targets/v5/) and *TargetScanHuman 6.1* (http://www.targetscan.org/).

### Re-sequencing of Candidate Genes

Candidate genes for the BTA5 and BTA27 FP QTL were re-sequenced in 24 and 12 HF animals, respectively. The animals were selected based on their genotypes for the most significantly associated SNP of the particular QTL. PCR primers were designed for the promoter regions (3000 bp and 1500 bp upstream of the transcription start site for *EPS8* and *GPAT4*, respectively), for all exons and intron-exon boundaries as well as for the 5′ and 3′ untranslated regions (UTR) (**Table S2**). The PCR products were sequenced using the BigDye® Terminator v1.1 Cycle Sequencing Kit (Applied Biosystems) on the ABI 3130×l Genetic Analyzer (Applied Biosystems). The *Phred/Phrap/Polyphred* software suite [34] was used for base calling, sequence alignment and polymorphism identification, and *consed* [35] was used for viewing.

### Genotyping of Selected Polymorphisms

Three previously proposed quantitative trait nucleotides (QTN) for FP, namely the ones responsible for the K232A-substitution within *DGAT1* [6], [7], the F279Y-substitution within *GHR* [9] and the S18N-substitution within *PRLR* [8] were obtained by TaqMan® genotyping analysis (Applied Biosystems Applera, Darmstadt, Germany). Potentially functional polymorphisms for the BTA5 and BTA27 QTL were genotyped in 2327 animals of our study population as well **(Table S3)**. Sporadically missing genotypes were imputed using default parameters of *Beagle* (version 3.2.1) [25]. The missing genotype rates were 3.1% for both the *DGAT1* and the *GHR* variant, 9.2% for the *EPS8* polymorphism ss319604833, 8.3% for ss410759404 and 11.3% for ss410758894 within *GPAT4*.

## Supporting Information

Figure S1 **Comparison of the pairwise pedigree** ***vs*** **. genomic relationship.** Pairwise pedigree *vs.* genomic relationship for the studied Holstein-Friesian population before (A) and after (B) the exclusion of 12 animals with inconsistencies.
(PDF)

Table S1 **Exon/intron boundaries of bovine genes for re-sequencing.**
(PDF)

Table S2 **Primers used for re-sequencing bovine genes.**
(PDF)

Table S3 **Primers and probes used for Taqman Genotyping Assay.**
(PDF)

Table S4 **Polymorphisms identified in bovine genes.**
(PDF)
