# Supplementary figures and images for: Identification and Dissection of Four Major QTL Affecting Milk Fat Content in the German Holstein-Friesian Population
Source: PLoS One. 2012 Jul 11;7(7):e40711. doi: 10.1371/journal.pone.0040711 (PMC3394711; doi:10.1371/journal.pone.0040711)

**A**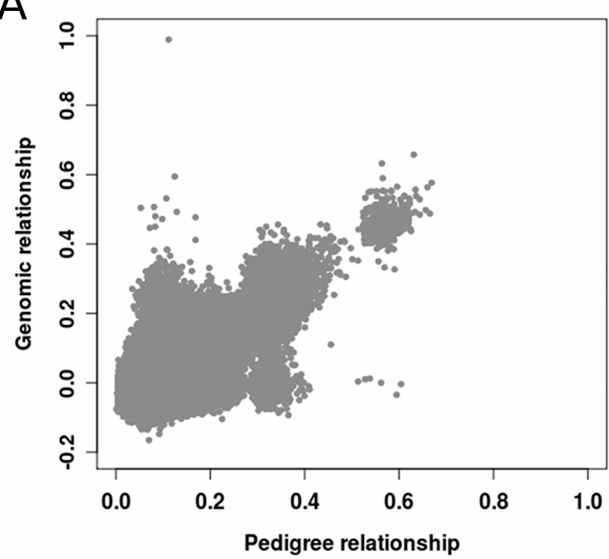**B**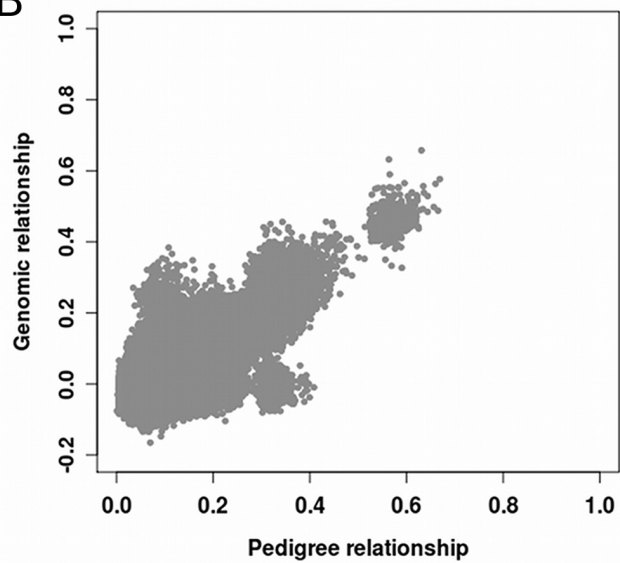

Supplement: Figure S1 — Comparison of the pairwise pedigree vs . genomic relationship. Pairwise pedigree vs. genomic relationship for the studied Holstein-Friesian population before (A) and after (B) the exclusion of 12 animals with inconsistencies. (PDF) [file pone.0040711.s001.pdf]
