# Supplementary material for: Identification and Dissection of Four Major QTL Affecting Milk Fat Content in the German Holstein-Friesian Population
Source: PLoS One. 2012 Jul 11;7(7):e40711. doi: 10.1371/journal.pone.0040711 (PMC3394711; doi:10.1371/journal.pone.0040711)
Supplement: Table S1 — Exon/intron boundaries of bovine genes for re-sequencing. (PDF) [file pone.0040711.s002.pdf]

Table S1 Exon/intron boundaries of bovine genes for re-sequencing

Gene	Exon	Length (bp)	Start position (bp)	End position (bp)
*EPS8*_transcript I	1	227	94553680	94553906
	2	74	94558442	94558515
	3	80	94674462	94674541
	4	74	94677617	94677690
	5	51	94688540	94688590
	6	68	94689953	94690020
	7	162	94691356	94691517
	8	150	94693298	94693447
	9	83	94696745	94696827
	10	137	94697410	94697546
	11	74	94700936	94701009
	12	533	94702355	94702887
*EPS8*_transcript II	1	227	94553680	94553906
	2	74	94558442	94558515
	3	80	94674462	94674541
	4	74	94677617	94677690
	5	68	94689953	94690020
	6	162	94691356	94691517
	7	150	94693298	94693447
	8	83	94696745	94696867
	9	137	94697410	94697546
	10	74	94700936	94701009
	11	127	94702355	94702481
	12	89	94704771	94704859
	13	75	94705230	94705304
	14	149	94712914	94713062
	15	184	94716329	94716512
	16	134	94721752	94721885
	17	100	94725123	94725222
	18	144	94726718	94726861
	19	223	94736955	94737177
	20	181	94745018	94745198
	21	130	94746416	94746545
	22	296	94748798	94749093
GPAT4	1	945	36211855	36212799
	2	70	36220385	36220454
	3	301	36220586	36220886
	4	75	36221569	36221643
	5	90	36222395	36222484
	6	94	36222622	36222715
	7	116	36223362	36223477
	8	56	36224201	36224256
	9	86	36224650	36224735
	10	129	36227886	36228014
	11	80	36228098	36228177
	12	658	36228357	36229014

The genomic position is according the UMD3.1 assembly.
