# Supplementary material for: Identification and Dissection of Four Major QTL Affecting Milk Fat Content in the German Holstein-Friesian Population
Source: PLoS One. 2012 Jul 11;7(7):e40711. doi: 10.1371/journal.pone.0040711 (PMC3394711; doi:10.1371/journal.pone.0040711)
Supplement: Table S2 — Primers used for re-sequencing bovine genes. (PDF) [file pone.0040711.s003.pdf]

Table S2 Primers used for re-sequencing bovine genes

Gene	Forward primer	Reverse primer	Region
EPS8	GATATTTCATGTCCTGACTAGACCA	CATATTCTCATCAAACCTGTAAGAAA	PROM
	TTTTTCAACTCCTGGTCCAAA	CATGGACAGTGAGCCTAGCA	PROM
	GTATTTTTCCCCCTCCCGTA	CTTTGTGAGCCCATGAAATG	PROM
	ATTGGGGACAGGAGGAGAAG	TGCCTTGAATGTGCTTTGAG	PROM
	CGACTGGGTGACTGAACTGA	AAATGAGATGGGCCTCGTTA	PROM
	TAACGAGGCCCATCTCATTT	TTCCGTCATCCTAACCAACC	PROM
	ATTCCTGGGAGTCCTGACCT	AAGTCGGACCGAAGTTCTGA	PROM
	CTCTTGCTCTCCTCCCCTTC	CTCCCGACACCAGTTGAAAG	EX1
	ATTCTGGCCTGGAGAAAACC	GGACACCTCAGTAGCAGCAA	EX2
	ACTCACCTGGGGTTTGATCC	GTCATTGGGGTTGATTGCTT	EX3
	TGGGTCAGGATCCGATAGTC	TCAGCCCATGATCAACAGAA	EX4
	AACCGCTGCCATTTCTTTTA	CGGTTTCACAATACCCATGA	EX5
	TGACCGTATTTGCAGTTCCA	TGTGCCTCAAGCTTCATTCT	EX5
	GTTGCCATTTCCTTCTCCAA	TTCCTCACTTCTTCCACCTCA	EX6
	GGCATCCTCTCTTTCCCATA	GACACGACTGAGCGACTTCA	EX7
	GCGCATAATCCCTCTTTCTG	TGCCTTGGGGTAGAAAAACA	EX8
	CCTTGTTTTTCTACCCCAAGG	TCAAACTCCCTCTTTGAGTGC	EX9
	TGGAAGGCTGGTACTTCAGC	TTGGCATAAAGGCAAAAATTG	EX10
	AGCCACAGGTATGCTAGTTGAA	CAGATGGCCACTATTGAATGA	EX11
	CTTGGAACTTTGCCCTCATC	CACAACAGACGCCAAAAGAA	EX12,EX13
	TGCATATAATCATCATCTTCTTGC	CTCTCCAAACCGTCTTCCTG	EX14
	TGGAAAGAGGGCAGACTAGG	GACGTCTCTGGTGGCTCAGT	EX15
	TACAGTCCATGGGGTCACAA	GGCATTTGCGCTGTTAGAAT	EX16
	CCCTGGCCATAGTTTGTTCA	TTCCCCACAAAACAAACACA	EX17
	ATATGGACCTGGGGTGGAAT	GGAAAAACCCGAATGAACCT	EX18
	TTTGCTGTGGATCTGCAAGT	GCTTCCCTAGTGGCTCAGTG	EX19
	GGGGTCTTGCCAAATGAATA	GGGTGCCAACAAAAGACAGT	EX20
	GTTTACCCCAGGATGCTGAA	TTCATTTTGCAGGCACACTT	EX21
	TCTTGCTTGACCTAAAGAGCA	CCCAAGGTCAAAACTTCTGC	EX22
	TGATCTGCAGCCTCACAGAC	GCAACAGAGCCGTGACAAAT	EX22
	TCCAGGGTGTAGCTTGCTTT	CCTTTTGCCAATGAGACACA	3'UTR
GPAT4	AAGTGAAAGCAAAGTCGGTCA	TGGTCACGTATGGATGTGAGA	PROM
	TCCAATGAACACCCAGGACT	ATTGCAGGGGAAAAGGAAAT	PROM
	CCACTGTTGCCCCATCTATT	GATGCTTCCTGAGACGTTCC	PROM
	TAACCGATTCACGGAGCATT	GGTTTGTCCACCACTACATGG	PROM
	TGGGTTAGTTTGTGCTGTGC	GCTCCTCTGGATGCTCCTC	PROM
	GACCTTGGGGATGAGAGGAG	TGCCAGAAAAAGCCTGATAA	PROM
	GGGCAGTATGAGCCATTCC	CAGAGGGTGAGAGCTGAAGG	PROM
	TCTTGGGCAGGAGATACAGG	TTGGCTAACGGCACTTCTCT	EX1
	TCTGATCTGGGGTGACTGG	GGCAGTAGCGGATGAGTACG	EX2
	CAAAAGACCCCACGTCACTA	GGGATCAATACGCCTCTGTC	EX3
	TTGGACCCTACTTCCTGCTG	GCTGCCGAGTCAGTTACCAT	EX4
	GGCAGCAGTGGTTAGATGGT	CCCAGGTCTGAAAGCACACT	EX5,6
	CGGTTCTTTGTAGGGTTTTCA	GCGCTTTATTCTATTGCTGGA	EX7
	TGTTCATTAGCCTGCCTTCA	CGGTCCTTTACCCCAGAGAT	EX8
	CTGGGGAGATGACAGGCTTA	GTGAGCCAAAGCAGAGGAGT	EX9
	TTGTTCACAGACCCATGCAG	AGCAACCGCCTCAGAAATC	EX10
	CTTGCAGGCAGAGGAGGAT	GTGGGGAGGGACTTTAATGG	EX11,12
	GTTCAAGGAGGAGCAGCAGA	ACAGGAACGAACCCCAAAC	3'UTR
	ACTGGTCCCCCAGCTCAGT	TTCCAGATCGTCTCCTACCC	3'UTR
	GATGTCTGCTGGGAAGAAGG	AGGAGATGCCGCTGCTAGT	3'UTR
	ACTGGCTTGACTGGGTTCAG	TGAGAAAACGAAAACCGACA	3'UTR
