# Supplementary material for: Identification and Dissection of Four Major QTL Affecting Milk Fat Content in the German Holstein-Friesian Population
Source: PLoS One. 2012 Jul 11;7(7):e40711. doi: 10.1371/journal.pone.0040711 (PMC3394711; doi:10.1371/journal.pone.0040711)
Supplement: Table S3 — Primers and probes used for Taqman Genotyping Assay. (PDF) [file pone.0040711.s004.pdf]

Table S3 Primers and probes used for Taqman Genotyping Assay

dbSNP ID	Gene	Primer	Probe
ss319604845	*EPS8*	F: GAATCTGGATTAGGACGTGCTGAT	VIC: CTGTATGGTATGCGTGTAAG
		R: GGGCAATGAATCTAAAAATTTGAAAAAGTT	FAM: TGTATGGTATGCATGTAAG
ss319604833	*EPS8*	F: GGCTGCCGCCAAGAC	VIC: CGCCCTGAGCCCAG
		R: CGCACGCCCAGTTCCT	FAM: CGCCCTAAGCCCAG
ss319604831	*EPS8*	F: GGTTCTAGAGATTTTGGAGCAGAAGA	VIC: CTGGTAGTTACTTAATAATTG
		R: TGACATCACAAGGCTTGAAATATTTGAAC	FAM: TCTGGTAGTTACTTAATTG
ss410759404	*GPAT4*	F: GCATCCGGAGTGTCCTAATGTTG	VIC: CTCCTCACTGTCCCCCAC
		R: GGCACACTCCAAGGAGAAGATG	FAM: TCCTCACTGACCCCAC
ss410758894	*GPAT4*	F: CGGCCAGCCCAGTGT	VIC: CTCTCCCTCACCCGCC
		R: ACAGGAACGAACCCCAAACC	FAM: CTCTCCCTTACCCGCC
