# Supplementary material for: Identification and Dissection of Four Major QTL Affecting Milk Fat Content in the German Holstein-Friesian Population
Source: PLoS One. 2012 Jul 11;7(7):e40711. doi: 10.1371/journal.pone.0040711 (PMC3394711; doi:10.1371/journal.pone.0040711)
Supplement: Table S4 — Polymorphisms identified in bovine genes. (PDF) [file pone.0040711.s005.pdf]

Table S4 Polymorphisms identified in bovine genes

Gene	dbSNP ID	Polymorphism	Localization	Amino acid
EPS8	ss319604828	C>T	PROM	
	ss319604829	A indel	PROM	
	ss319604830	A>G	PROM	
	ss319604831	TAA indel	PROM	
	ss319604832	A>G	PROM	
	ss319604833	C>T	PROM	
	ss319604834	G>T	EX1 (5'UTR)	
	ss319604835	C>T	INT1	
	ss319604836	GT_indel	INT8	
	ss319604837	C>T	INT8	
	ss319604838	A>G	INT8	
	ss319604839	C>T	INT9	
	ss319604840	C>T	INT10	
	ss319604841	C>T	INT11	
	ss319604842	A indel	INT15	
	ss319604843	A>C	INT15	
	ss319604844	A>G	INT16	
	ss319604845	C>T	EX18	M599T
	ss319604846	A>C	INT19	
	ss319604847	C>T	INT20	
GPAT4	ss410758869	A>G	PROM	
	ss410758870	A>G	PROM	
	ss410758871	C>G	PROM	
	ss410758872	A>G	PROM	
	ss410758873	A>G	PROM	
	ss410759404	GA>-T	PROM	
	ss410758875	C>T	PROM	
	ss410758876	G>T	PROM	
	ss410758877	A>G	PROM	
	ss410758878	C>T	PROM	
	ss410758879	C>T	EX1	T24
	ss410758880	C>T	EX3	D114
	ss410758881	C>T	INT3	
	ss410758882	G>T	INT3	
	ss410758883	A>G	INT4	
	ss410758884	A>G	INT6	
	ss410758885	C>T	INT7	
	ss410758886	C>G	INT8	
	ss410758887	C>G	INT9	
	ss410758888	A>G	INT9	
	ss410758889	C>T	INT10	
	ss410758890	A>G	INT11	
	ss410758891	G>T	INT11	
	ss410758892	C>T	INT11	
	ss410758893	A>G	3'UTR	
	ss410758894	A>G	3'UTR	
	ss410758895	A>C	3'UTR	
	ss410758896	A>G	3'UTR	
	ss410758897	C>T	3'UTR	
	ss410758898	A>G	3'UTR	
